# Dephenolization Methods, Quality Characteristics, Applications, and Advancements of Dephenolized Cottonseed Protein: Review

**DOI:** 10.3390/foods14040628

**Published:** 2025-02-13

**Authors:** Xuegang Huang, Yumeng Hu, Zhenyuan Li, Bo Jiao, Xiaojie Ma, Qin Guo, Qiang Wang

**Affiliations:** Institute of Food Science and Technology, Chinese Academy of Agricultural Sciences, Key Laboratory of Agro-Products Processing, Ministry of Agriculture, Beijing 100193, China; huangxuegang2022@163.com (X.H.);

**Keywords:** cottonseed meal, cottonseed protein, dephenolization, preparation methods, quality characteristics, applications

## Abstract

Dephenolized cottonseed protein is a high-protein product obtained through the further dephenolization of cottonseed meal or by removing the lint and shell of cottonseed, extracting the oil at a low temperature, and subsequently eliminating toxic substances (gossypol). This paper presents a review of the latest advancements in the dephenolization methods, quality characteristics, and application domains of dephenolized cottonseed protein. It focuses on enhanced dephenolization methods, and summarizes the composition, structural characteristics, functional properties, and recent research developments. Additionally, it identifies challenges, opportunities, and new directions for future research on dephenolized cottonseed protein, which will contribute to advancing the field of dephenolized cottonseed protein research.

## 1. Introduction

Cotton is distributed globally, with a planted area of 70.3 million acres in 2023, with China accounting for 9.8% of this. Moreover, its production in China amounted to 5.618 million tons, constituting 22.7% of the global production [1,2]. Currently, 88.8% of China’s cottonseed is utilized for oil extraction and reprocessing, and the production volume of cottonseed meal was 4.116 million tons in 2023 [3], accounting for 27.4% of the world. The crude protein content of cottonseed meal ranges from 45 to 50% [2], making it a valuable source of protein. Gossypol, cycloallyl fatty acids, tannins, phytic acids, and non-starch polysaccharides are contained in cottonseed meal, among which free gossypol is the most important anti-nutrient factor, with a strong toxicity and high content [4]. Because of the limitation of its free gossypol content, 98% of cottonseed meal in China is used in the feed industry (accounting for 3.9% of feed meal), and less than 2% is utilized in the extraction of vegetable proteins, production of food additives, and preparation of environmentally friendly materials. Concurrently, the cottonseed protein industry faces several challenges that impede its development, such as a low production efficiency, poor product quality stability, high production costs, and low industrial efficiency. Consequently, reducing or removing gossypol from cottonseed protein to enhance its quality is of significant importance.

**Figure 1:**
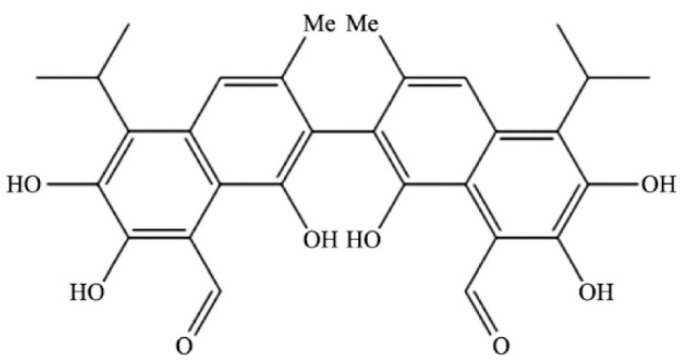
Chemical structure of gossypol.

Gossypol (Figure 1) is a polyphenolic compound produced by cotton glands. It is yellow and crystalline, and exists in cottonseed protein in the form of bound gossypol and free gossypol. Free gossypol is toxic and can induce reproductive inhibition and hepatotoxicity in livestock, poultry, and humans, to varying degrees [5]. Based on its free gossypol content, cottonseed protein can be categorized into ordinary cottonseed protein (generally derived from cotton seeds and directly squeezed through an oil meal, 1200–5000 mg/kg) and dephenolized cottonseed protein (for feeding, ≤1200 mg/kg) [6]. The feed hygiene standard stipulates that the free gossypol content must be ≤400 mg/kg [7], and there is no industry definition for food-grade dephenolized cottonseed protein. The Protein Advisory Group of the World Health Organization (WHO), Food and Agriculture Organization of the United Nations (FAO), and United Nations Children’s Fund (UNICEF) recommend that the free gossypol content in edible cottonseed products should be ≤600 mg/kg. The Food and Drug Administration (FDA) stipulates that the free gossypol residue in cottonseed products used as ingredients should be ≤450 mg/kg. However, China has not yet established standards for dephenolized cottonseed protein in food and other fields.

Currently, China is the sole global producer of dephenolized cottonseed protein on a mass scale, with market products primarily consisting of 50%, 55%, 60%, and 65% dephenolized cottonseed protein concentrations. The production volume reached 616,200 tons in 2023, constituting 14.97% of China’s cottonseed meal output, with sales amounting to USD 260 million [8]. The production of dephenolized cottonseed protein in China is predominantly concentrated in Xinjiang, particularly for high-protein (60% protein) raw materials. The principal manufacturers include Chenguang Biotechnology Co., Ltd. (Xinjiang, China), Xinjiang Jinlan Plant Protein (Xinjiang, China), Xinjiang Taikun Protein (Xinjiang, China), Xinsai Co., Ltd. (Xinjiang, China), and Tiankang Biotechnology (Xinjiang, China). Existing research on dephenolized cottonseed protein at home and abroad mainly focuses on gossypol removal, protein extraction, and functional evaluation. Additionally, the hydrolysate of cottonseed protein and its application as a bioactive peptide with antioxidant, antibacterial, and immunomodulatory functions have also been reviewed. In actual production, researchers are concerned about the amount of dephenolized cottonseed protein added as feed and the feasibility of its application in food. Over the past decade, notable advancements have been made in the removal of free gossypol from dephenolized cottonseed protein. However, its structural characteristics, functional properties, and applications in the fields of feed and food have not yet been comprehensively and systematically summarized. For this reason, this paper provides a detailed review of the dephenolization methods for cottonseed protein, the quality characterization of dephenolized cottonseed protein, and its application areas to provide scientific support for expanding the application areas of dephenolized cottonseed protein and enhancing its added value.

## 2. Dephenolization Methods for Cottonseed Protein

**Figure 2:**
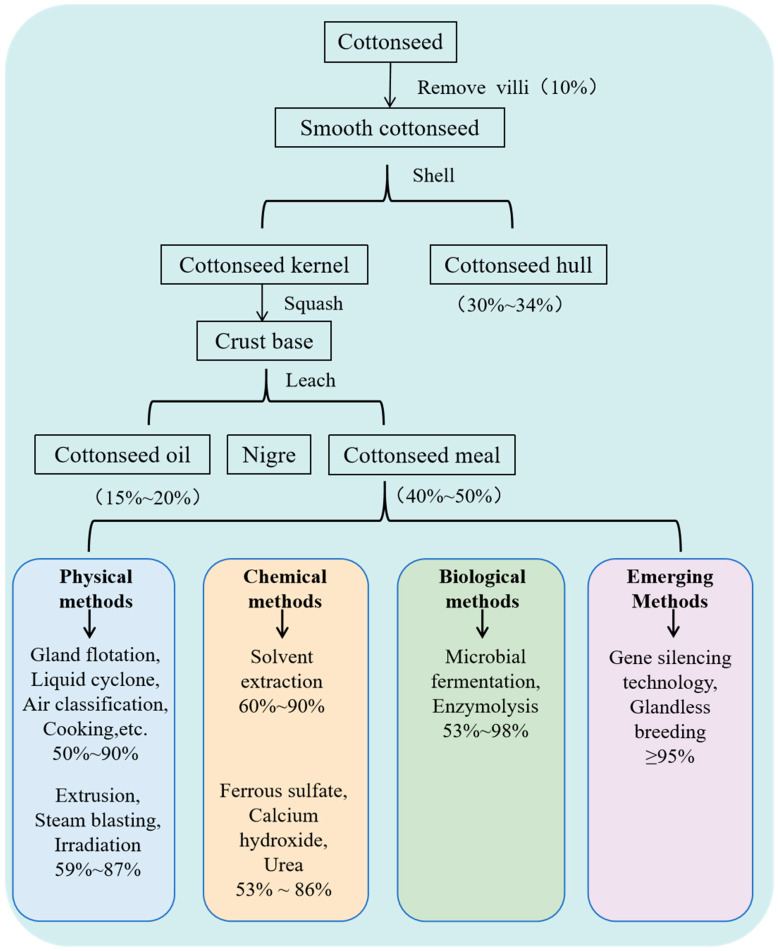
Dephenolization methods for cottonseed protein.

**Figure 3:**
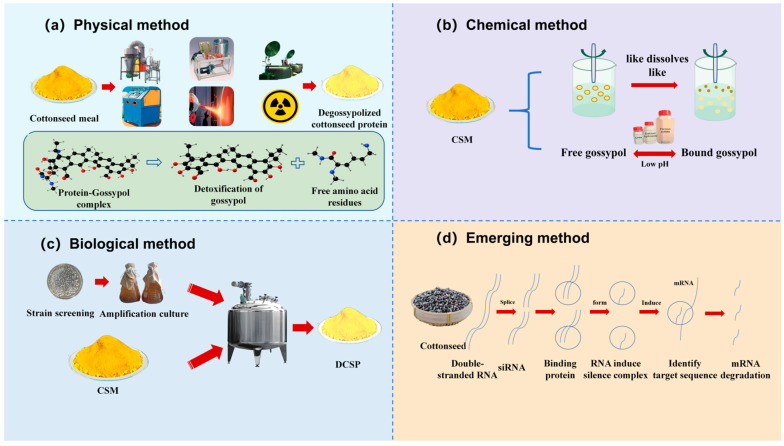
Mechanism of dephenolization of cottonseed protein by different dephenolization methods. Note: (**a**) the diagram shows the common physical dephenolization methods, from left to right, air classification, glandular flotation, cooking, steam blasting, high-energy electron beam, irradiation, and below is the diagram of dephenolization of protein-gossyphenol complex; (**b**) the figure shows the dephenolization of organic reagents and chemical reagents, respectively. The main principle of dephenolization of organic reagents is “like dissolves like”. Free gossypol and organic solvents are both weakly polar, and free gossypol is more soluble in organic solvents to achieve dephenolization. Urea, calcium hydroxide, and ferrous sulfate are dephenolized by converting free gossypol to bound gossypol. In addition, at a low pH, partially bound gossypol converts to free gossypol; (**c**) the figure shows the microbial fermentation method. The main principle is that microorganisms produce some enzymes that can decompose or transform free gossypol into gossypol binding enzymes during their growth, reproduction, and metabolic activities in raw media, so as to achieve the purpose of removing free gossypol. The specific mechanism of dephenolization is still unclear; and (**d**) the figure shows the gene silencing technique, which disables the expression of genes associated with cottonseed glands in cottonseed, thereby achieving source dephenolization.

Dephenolized cottonseed protein is a high-protein (>50%) product obtained through the further dephenolization of cottonseed meal or by removing the fluff and shell of cottonseed, extracting the oil at a low temperature once, and subsequently removing toxic substances (gossypol, ≤1200 mg/kg) (Figure 2). Currently, the primary dephenolization methods encompass physical, chemical, and microbiological approaches (Figure 3) [9].

### 2.1. Physical Methods

Physical methods achieve dephenolization by altering the physical and chemical structures of cottonseed protein. These methods primarily include thermal treatment processes, such as gland flotation, liquid cyclones, air classification, and cooking [10]. Gland flotation technology utilizes the density differential between gossypol-containing glands and nuclear tissue to separate the glands from a solvent slurry with a density lower than that of the glands through agitation flotation. Liquid cyclone technology involves suspending cottonseed in a low-moisture solvent mixture, dispersing gossypol-containing glands through a colloid mill, and subsequently separating them from the meal by gravity or sedimentation. This process can reduce free gossypol to below 400 ppm; however, the cost of hydrocyclone limits its industrial application. Air classification technology is more economical and advantageous than the hydrocyclone process, but it has not yet been implemented for industrial production [11]. Steam treatment has also been demonstrated as an effective method for removing gossypol from cottonseed protein. Most of the free gossypol in cottonseed meal can be removed by cooking. Nevertheless, owing to the high moisture content of steamed cottonseed meal, its industrial application is subject to certain limitations.

In recent years, several novel physical technologies, including extrusion puffing, steam explosion, and irradiation, have garnered attention from researchers. Extrusion puffing technology (puffing temperature of 120 °C) can remove 68% of the free gossypol in cottonseed meal; however, issues regarding the unstable quality of cottonseed protein meal post-puffing persist [12]. Steam explosion technology (steam pressure of 2.0 MPa, 30% water to material ratio, and 30 s holding pressure) applied to cottonseed meal resulted in a free gossypol removal rate of 87% (content reduced to 85.0 mg/kg) [13]. The free gossypol removal rate of cottonseed meal treated with a high-energy electron beam (40 kGy) was 82.37%, while gamma ray treatment achieved a removal rate of 59.16% [14]. These studies indicate that, compared to traditional dephenolization methods, the technical parameters for extrusion, steam explosion, and irradiation require further optimization to enhance the removal rate of free gossypol from cottonseed protein. Among them, steam explosion has emerged as a promising processing technology owing to its green, environmentally friendly, economical, and efficient performance. However, in practical production, this tends to cause cottonseed protein agglomeration, and issues such as uneven heating remain unresolved. The utilization of irradiation technology is increasing because of its simplicity, rapidity, and cost-effectiveness. However, owing to the side effects associated with high radiation doses and the limited extent of free gossypol removal, this dephenolization process requires further exploration and investigation. Additionally, the influence of various factors on the efficacy of physical dephenolization methods and the quality of dephenolized cottonseed protein warrants further consideration.

### 2.2. Chemical Methods

Among the chemical dephenolization methods, the removal of free gossypol using organic solvents is the most widely utilized method in industrial production. The principle involves employing a solvent that interacts with the glands in cottonseed or cottonseed meal to dissolve the gossypol. The solid–liquid interface produces a strong detergent effect on the solid, causing the solute (gossypol) to diffuse into the bulk liquid phase through molecular diffusion, thereby achieving gossypol removal. Commonly employed organic solvents include n-hexane, pentane, hexane, heptane, and octane. As environmental awareness has increased, methanol, ethanol, propanol, butanol, isopropanol, acetone, and their combined solvents have been used for gossypol removal. Among these, the most frequently employed reagent in industrialization is methanol, with a gossypol removal rate of 80% or higher.

A methanol–water mixed solvent (methanol volume fraction 80–90%) can reduce the free gossypol content in cottonseed meal to below 400 mg/kg after a single dephenolization at a solid–liquid ratio of 1:0.5–0.65. Concurrently, this process facilitates increased raffinose extraction, resulting in an overall yield increase [15]. To address the insufficient dephenolization achieved with a single reagent, a mixed reagent of methanol–ethanol (1:1) was employed for cottonseed protein dephenolization (solvent volume fraction 90%, temperature 55 °C, and time 25 min). After three dephenolization cycles, the free gossypol content decreased to 157 mg/kg [16], which was significantly lower than the gossypol content required for industrial production (below 400 mg/kg). In the presence of phosphoric acid, 90% acetone or ethanol (material–liquid ratio 1:10 and time 2.0 h) removed from 90% to 95% of the total gossypol from cottonseed protein, with ethanol demonstrating a higher dephenolizing effect and rate than acetone [17]. Isopropanol has also been identified as an effective solvent for dephenolization, serving as an alternative to hexane [18]. Research has shown that the use of an acidic ethanol solvent to remove free gossypol (down to 340 mg/kg) results in a readily digestible cottonseed protein powder with a 53.8% protein content, which can potentially serve as a substitute for fishmeal in feed applications [19].

In recent years, chemical reagents such as ferrous sulfate, calcium hydroxide, and urea have been utilized for cottonseed meal dephenolization, which is based on the principle of converting the free gossypol in cottonseed protein into nontoxic bound gossypol through chemical reactions, thereby achieving dephenolization. The ferrous ions (Fe^2+^) in ferrous sulfate can combine with free gossypol to form coordination complexes. When cottonseed meal was subjected to heat treatment at 80 °C in a closed environment for 1 h with 16% ferrous sulfate, the removal rate of free gossypol was 84% [20]; however, ferrous sulfate dephenolization also causes the problem of a black coloration and poor palatability of cottonseed meal. Calcium hydroxide can function as an alkaline substance that combines with the acidic hydroxyl groups in free gossypol to achieve detoxification. When cottonseed cake was immersed in 3% calcium hydroxide for 24 h, the removal rate of free gossypol reached 85.87% [21]. Urea can react with free gossypol to produce a Schiff’s base, which can convert free gossypol into bound gossypol, effectively compensating for the poor palatability caused by ferrous sulfate dephenolization, albeit with a lower removal rate (53.64%) [22]. Cottonseed meal treated with ferrous sulfate, calcium hydroxide, and urea exhibits a propensity to increase the free gossypol content as the pH decreases; thus, it is imperative to maintain the pH at neutral or slightly alkaline levels. Additionally, free gossypol can combine with some of the free amino groups of lysine and arginine through covalent bonds to produce bound gossypol, thereby reducing the gossypol content [23].

The aforementioned analysis demonstrates that the solvent removal of free gossypol from cottonseed protein is the most efficacious method in industrial processing. The rate of free gossypol removal when utilizing common alcohol solvents ranges from approximately 60 to 90% under the following conditions: a 50100% concentration, a 1:0.524 solid–liquid ratio, 14 dephenolization iterations, a 0.53 h dephenolization time, and a 3560 °C dephenolization temperature. As the solvent concentration, solid–liquid ratio, and number of dephenolization cycles increased, the removal rate of free gossypol initially increased and then gradually plateaued. Furthermore, the utilization of solvent compounds can marginally enhance the free gossypol removal rate to a certain extent; however, the improvement effect is also limited, and the impacts of different solvents and their compounds on the quality of dephenolized cottonseed protein necessitate a systematic analysis. At present, the degree of free gossypol removal is insufficient to meet the diverse applications of cottonseed protein across various fields, necessitating the exploration of novel methods that are straightforward, environmentally friendly, efficient, cost-effective, and employ reusable solvents. Additionally, it was observed that controlling the hull content of cotton kernels (<2%), the particle size of cotton germ powder, and softening cotton germ could further reduce the free gossypol content; however, the optimal relationship among these factors remains unclear.

### 2.3. Biological Methods

Biological dephenolization has become a hot topic in current research because of its ability to effectively reduce gossypol content without being absorbed by the digestive system. This process involves microbial fermentation and enzymatic hydrolysis. Microorganisms belonging to the genera *Saccharomyces*, *Aspergillus*, *Trichoderma*, *Rhizopus*, and *Bacillus* (*Pseudomonas tropicalis* [24], *Aspergillus niger* [25], *Bacillus subtilis* [26], *Lactobacillus* [27], etc.) demonstrate efficacy in the degradation of free gossypol in cottonseed meal. The fundamental principle involves microorganisms producing enzymes capable of decomposing or converting free gossypol into bound gossypol during their growth, reproduction, and metabolic activities in a raw material culture medium, thereby achieving free gossypol removal [28]. Currently, *Bacillus* sp. exhibits a higher removal rate for free gossypol in cottonseed meal, whereas *Lactobacillus* and *Saccharomyces cerevisiae* have garnered considerable attention from researchers because of their relatively high safety profiles, which have become the focus of future research on microbial fermentation dephenolization. The aforementioned methods remain in the laboratory research phase, and the mechanism of microbial dephenolization has not yet been fully elucidated. Mageshwaran et al. [29] utilized *Candida tropicalis* ZD-3 (inoculation amount 15%, substrate moisture 70%, fermentation temperature 30 °C, and time 48 h) to reduce the free gossypol content in cottonseed meal from 341.0 mg/kg to 69.9 mg/kg, achieving a removal rate of 79.5%. Under identical fermentation conditions, the inoculation amount of *Aspergillus niger* was 8%, resulting in a reduction in the free gossypol content in cottonseed meal from 549.1 mg/kg to 81.5 mg/kg, with a removal rate of 85.1% [30]. *Bacillus subtilis* M underwent induced mutagenesis, and the resultant mutant strain M-15 obtained by rescreening was fermented (carbon source 4% corn flour, substrate moisture 45–50%, bacterial agent 1.0 × 10^9^ CFU/t, and time 14 days), which reduced the free gossypol content in cottonseed meal from 980 to 63.7 mg/kg, achieving a removal rate of 93.5% [26]. This method demonstrated a superior dephenolization efficacy.

*Bacillus coagulans* combines the characteristics of both lactic acid bacteria and *Bacillus*, possessing the ability to produce lactic acid and spores. The free gossypol content in cottonseed meal can be reduced from 923.8 mg/kg to 167.9 mg/kg through the application of *Bacillus coagulans* (carbon source 2% corn flour, 1% bran, inoculation amount 15%, substrate moisture 50%, fermentation temperature 40 °C, time 52 h, and 0.3–0.8% metallic iron), resulting in a removal rate of 81.83% [31]. *Pediococcus pentosaceus* is a lactic acid bacterium characterized by rapid proliferation and a high safety. *Pediococcus pentosaceus* (inoculation amount 2%, substrate moisture 50%, fermentation temperature 37 °C, and time 7 days) can remove 87.1% of free gossypol from cottonseed meal [32]. Meanwhile, mixed-bacteria fermentation has also garnered significant attention from researchers, owing to its potential to enhance the fermentation flavor and improve the nutritional value of cottonseed protein. However, the dephenolization process of mixed multi-bacterial fermentation requires further investigation [33]. For instance, the removal rate of free gossypol from cottonseed meal reached 79.44% during the combined anerobic fermentation of *Saccharomyces cerevisiae* and *C. tropicalis* (30 °C and 48 h) [29]. In contrast, the removal rate of free gossypol from cottonseed meal was only 60.0% with the mixed fermentation of *Saccharomyces cerevisiae* and *Staphylococcus tropicalis* (inoculation ratio 1:1, substrate moisture 75%, fermentation temperature 32.5 °C, and time 54 h) [34]. It was determined that the microbial fermentation method was most effective under the following conditions: 2–8% inoculum of bacterial solution, 45–75% substrate moisture, 30–40 °C fermentation temperature, and 2–7 days fermentation time, with the free gossypol removal rate ranging from 53.0% to 93.5% and the protein content increasing by 4–15%.

In addition to screening for more efficient and safe strains for the removal of free gossypol from cottonseed meal, researchers have also achieved significant results for the degradation of gossypol in cottonseed meal using enzymatic (laccase, peroxidase, and polyphenol oxidase) methods. Studies have shown that the addition of Cot A laccase from Bacillus licheniformis can achieve from 87% to 98% FG degradation in defatted CSM within 2 h [35]. *Bacillus subtilis* BJ-1 (2% inoculum, fermentation temperature 37 °C, and time 48 h) demonstrated the capacity to remove 66.7% of the free gossypol from cottonseed meal [36]. Furthermore, the addition of 0.1% (*w*/*v*) papain as a fermentation adjuvant not only enhanced the hydrolysis degree of cottonseed protein, but also improved its antioxidant function and in vitro digestibility; however, its effect on the free gossypol removal rate was not statistically significant. Additionally, the fermentation pH (4–6) of cottonseed meal and mineral supplementation (e.g., Mn^2+^, Cu^2+^, Fe^2+^, and Mg^2+^) were observed to influence the dephenolization of cottonseed meal.

The biological dephenolization method is considered to be a sustainable approach that can be used in conjunction with physical methods, chemical solvent methods, or other approaches to effectively remove gossypol to acceptable levels. However, several of the following factors must be taken into consideration: (1) the selected screening strains or enzymes must be safe, non-toxic, or have been extensively studied to confirm that no harmful substances are produced during the fermentation process and can be added to feed and food; (2) the quantity of screening strains or enzymes should be strictly regulated under the reaction conditions and comply with the relevant standards for feed or food applications; and (3) the use of screening strains or enzymes should not significantly impact the quality of cottonseed protein. Furthermore, among the existing biological dephenolization methods, enzymatic dephenolization demonstrates a superior efficacy compared with microbial fermentation; however, the fermentation conditions require further optimization. In conclusion, the bio-fermentation method offers advantages, such as mild reaction conditions, a low energy consumption, an increased protein content, an enhanced essential amino acid content and digestibility, and an improved flavor, indicating significant developmental potential. Nevertheless, its dephenolization efficacy is inferior to that of chemical methods, and it is characterized by prolonged reaction times and relatively high costs. Future research should focus on elucidating the mechanism of gossypol removal from biodegradable cottonseed meal and evaluating the safety of dephenolized cottonseed meal.

### 2.4. Emerging Methods

In an effort to reduce or completely eliminate gossypol at its source, researchers from Texas A&M University used RNAi-mediated gene silencing technology to decrease the gossypol content in cottonseed by 97% [10]. Gao et al. [37] (2020) utilized biotechnology to silence the CGP1 and GoPGF genes associated with cottonseed glands, thereby achieving the controlled synthesis of gossypol in cotton. Researchers from the New Mexico Agricultural Experiment Station developed the glandless cotton variety NuMex COT 15 GLS [38]; however, this approach continues to present challenges regarding transgenic safety, which remain difficult to control.

## 3. Quality Characteristics of Dephenolized Cottonseed Protein

### 3.1. Composition

Dephenolized cottonseed protein exhibits varying colors, ranging from light yellow to brown, depending on the oil extraction process, typically appearing light yellow or yellow. The protein quality is inversely correlated with the degree of protein denaturation; the lower the degree of protein denaturation, the better the quality. Dephenolized cottonseed protein comprises moisture, ash, crude protein, crude fiber, total sugar, protein composition, subunit composition, and content (Table 1 and Table 2). Moisture is an indispensable constituent of the dephenolized cottonseed protein system, affecting not only the protein conformation, but also its functional properties such as viscosity, solubility, and gelation. The moisture content of dephenolized cottonseed protein is lower than that of bulk oilseed protein. Ash consists of water-insoluble minerals and organic components, including trace elements, such as calcium and phosphorus. A high ash content in feed may adversely affect the digestion and absorption of dephenolized cottonseed protein in animals and reduce its palatability. The crude fiber content of dephenolized cottonseed protein exceeds that of soybean protein, primarily because of the presence of cotton hulls. This indicates that existing dephenolized cottonseed protein contains a certain proportion of cotton hulls, suggesting that the cottonseed dehulling rate requires further improvement to achieve a competitive advantage over bulk oilseed proteins. The protein content of dephenolized cottonseed protein exhibits a high market share of 57.1–61.50% (based on the feed standard protein coefficient of 6.25) and 48.2–52.40% (based on the food standard protein coefficient of 5.3), which is lower than that of soybean and peanut protein but higher than that of sunflower protein. The crude fiber content ranges from 4.20 to 5.85%, which is higher than that of soybean protein, but lower than that of peanut and sunflower proteins. Foods with a crude fiber content exceeding 2% are considered to be dietary fiber foods, and soy protein powder requires a crude fiber content of ≤5.00% [39]. Therefore, the crude fiber content needs to be further reduced in order to use dephenolized cottonseed protein in the food industry.

The primary component of cottonseed protein is globulin, the globulin content is about 90%90% of the total protein, followed by glutenin. Based on the sedimentation coefficient, globulin is categorized into 2S, 7S, and 12S fractions. The 2S protein, located outside the protein body, accounts for 30%, whereas 7S and 12S collectively represent 60% [46]. Similarly, soy protein and peanut protein predominantly consist of globulin, with the 7S and 11S fractions of soybean protein constituting 87% [47] and the 7.8S and 14S fractions of peanut protein accounting for 79% [48,49]. The 11S fraction accounts for 70% of sunflower seed protein, whereas cottonseed protein exhibits a higher 2S content than bulk oilseed proteins. Variations in the composition and content of different oilseed proteins contribute to distinct functional properties. Approximately 60% of cottonseed protein consists of molecular mass subunits ranging from to 22 to 27 kDa, with 42 kDa and 48 kDa subunits, each accounting for 14.3% [50], similar to peanut protein. Soybean protein subunits are predominantly concentrated in the middle molecular weight range, whereas the subunit content of sunflower seed proteins has not yet been reported [51]. The above analysis indicates significant variability in the number, molecular weight, and content of subunits among the different oilseed proteins. Further investigation is necessary to elucidate the relationships between these subunits and the functional properties of the proteins, particularly the association between cottonseed and sunflower seed protein subunits and their respective functional characteristics.

**Table 2:** Comparative analysis of components and subunits of dephenolized cottonseed protein and bulk oilseed protein.

Type	Protein Composition and Content (%)	Subunit Composition and Content (%)	References
Cottonseed protein	2S, 30% 7S and 12S, 60%	14 kDa, 34.1%	[46]
		18 kDa, 26.5%	
		26 kDa, 8.2%	
		33 kDa, 2.6%	
		42 kDa, 14.3%	
		48 kDa, 14.3%	
Soy protein	2S, 8% 7S, 35% 11S, 52% 15S, 5%	Acidic subunit, 19.5–23.9%	[52,53]
		Basic subunit, 9.1–13.5%	
		52 kDa, 5.3–8.3%	
		72 kDa, 2.4–6.5%	
		76 kDa, 2.4–8.7%	
Peanut protein	2S, 21% 7.8S, 6% 14S, 73%	15.5 kDa, 4.5–11.9%	[54]
		17 kDa, 6.9–13.6%	
		18 kDa, 5.9–11.7%	
		23.5 kDa, 18.7–27.4%	
		35.5 kDa, 5.7–19.2%	
		37.5 kDa, 6.9–17.4%	
		40.5 kDa, 6.8–16.0%	
		61 kDa, 13.5–25.3%	
Sunflower seed protein	2S and 7S, 30% 11S, 70%	/	[55]

### 3.2. Nutritional Properties

Amino acids are fundamental components of proteins and are essential nutrients for the body. They play crucial roles in building and repairing body tissues, synthesizing enzymes and hormones, and maintaining the normal functions of the immune and nervous systems. The amino acid composition of dephenolized cottonseed protein is considered to be more favorable, its histidine content is higher than that of the bulk oilseed protein, and the composition ratio of lysine and methionine is comparable to that of the ideal protein model. There is a gap in the total amount of essential amino acids compared to that of bulk oilseed proteins (Table 3). The arginine content is higher than that of soybeans and comparable to that of peanut protein, which is conducive to the release of growth hormones and promotes the growth and development of the body.

Vitamins are a class of trace organic compounds essential for maintaining normal physiological functions in humans and animals, obtained through dietary intake. These compounds play crucial roles in growth, metabolism, and physiological development. Vitamin E (tocopherol) ranged from 1810 to 2280 μg/100 g. Notably, γ-tocopherol constituted 80.7–86.8% of the vitamin E content, suggesting that it is a characteristic nutrient of dephenolized cottonseed protein (Table 4).

The nutritional evaluation indexes of dephenolized cottonseed protein, including its efficacy ratio (2.0), digestibility (61%), raw material price (62%), and net protein utilization (41–47%), are higher than those of cereal protein (efficacy ratio 1.6–2.0, digestibility 35–45%, raw material price 53–70%, and net utilization 40–45%), but slightly lower than those of legume protein (efficacy ratio 1.7–2.1, digestibility 80–90%, raw material price 58–64%, and net utilization 50–61%). In comparison to soy protein, dephenolized cottonseed protein has no beany or other off-flavors, possesses a mild taste, and can complement the flavors of other food ingredients [56]. Additionally, dephenolized cottonseed protein contains low levels of indigestible oligosaccharides that do not induce flatulence. The American Agricultural Research Center administered dephenolized cottonseed protein products to malnourished young children. After 4 or 5 months, the nutritional status of the children exhibited significant improvement.

### 3.3. Structural Characteristics

The secondary structure of dephenolized cottonseed protein primarily refers to the specific hydrogen-bonding pattern between the amino groups in its main chain. This structure is important for understanding its function and nutritional value. When the α-helix content is elevated, the protein exhibits a less compact structure, and β-sheet is a configuration in which multiple peptide chains or several peptide segments of a peptide chain are arranged in parallel, with adjacent backbone chains maintained by hydrogen bonds, reflecting, to a certain extent, the degree of protein orderliness. β-turn is a 180° fold structure comprising four amino acid residues; random coil denotes an irregular peptide chain conformation in the secondary structure of the protein, indicating the extent of disorder in the protein structure.

The secondary structure of dephenolized cottonseed protein is predominantly characterized by β-sheet (39.5–39.8%), followed by β-turn (20.8–22.2%), random coil (19.9–20.9%), and α-helix (17.8–19.7%). Compared to bulk oilseed proteins (Table 5), the structure of the four basic proteins exhibits a uniform distribution in the dephenolized cottonseed protein. The α-helix content is observed to follow the order of peanut protein > dephenolized cottonseed protein > soybean protein > sunflower protein. The β-sheet content follows the order of sunflower protein > dephenolized cottonseed protein > soybean protein > peanut protein. The β-turn content is ranked as follows: sunflower protein < dephenolized cottonseed protein < peanut protein < soybean protein. The random coil content follows the order of sunflower seed protein > peanut protein > dephenolized cottonseed protein > soybean protein. The α-helix and β-sheet structures are relatively compact, resulting in limited unfolding of the peptide chain, which consequently restricts the protein solubility. Both these random coil and α-helix structures positively contribute to protein gel formation. However, the low random coil content of dephenolized cottonseed protein affects its protein gel formation capacity, thereby imposing certain restrictions on its application in food and other fields.

The two free sulfhydryl groups in proteins undergo oxidation to form disulfide bonds; conversely, disulfide bonds can be reduced to free sulfhydryl groups. However, the protein structure becomes less compact after the formation of sulfhydryl groups [57]. The free sulfhydryl groups and disulfide bond content are closely associated with the stability of the tertiary structure [58]. Free sulfhydryl groups exposed to proteins exhibit a strong polarity and hydrophilicity, and an increase in free sulfhydryl group content contributes to an improved protein solubility, while an increase in disulfide bond content contributes to the formation of a protein network structure and an enhanced protein gel strength [59,60]. The free sulfhydryl group content of dephenolized cottonseed protein ranges from 3.60 to 4.41 μmoL/g, and the disulfide bond content ranges from 35.28 to 39.72 μmoL/g. In comparison to bulk oilseed proteins, dephenolized cottonseed proteins have lower contents of free sulfhydryl groups and disulfide bonds than soybean and sunflower seed proteins. However, the free sulfhydryl group and disulfide bond contents of dephenolized cottonseed protein are not substantially different from those of peanut protein, which is attributed to the distinct protein structure characteristics, growth environment, and processing conditions.

**Table 5:** Comparison of structural parameters between dephenolized cottonseed protein and bulk oilseed protein.

Type	*α*-Helix (%)	*β*-Sheet (%)	*β*-Turn (%)	Random Coil (%)	Free Sulfhydryl Group (μmol/g)	Disulfide Bond (Chemistry) (μmol/g)	References
Dephenolized cottonseed protein	17.8–19.7	39.5–39.8	20.5–22.2	19.9–20.9	3.6–4.41	35.28–39.72	Laboratory self-tests
Soy protein	13.6	35.8	32.7	17.9	4.8	52.2	[61]
Peanut protein	25.9	29.1	23.7	21.3	4.2	35.8	[62]
Sunflower seed protein	14.3	36.7	32.2	16.8	6.7	50.1	[63]

### 3.4. Functional Characteristics

Dephenolized cottonseed protein is considered to be a potential source of protein for animal and human consumption. Consequently, its functional properties during processing, storage, and consumption significantly influence the quality, flavor, appearance, and taste of feed, and in particular, food products [64].

#### 3.4.1. Solubility

Protein solubility (Table 6) refers to the degree of protein dispersion in water; proteins with a high solubility generally exhibit superior functional properties. The protein nitrogen solubility index (NSI) is commonly used to quantify protein solubility [65]. Highly soluble proteins typically demonstrate enhanced gelling, emulsifying, foaming, oil absorption, and lipoxygenase activity, facilitating their widespread application in the food industry [66]. In comparison to proteins with a similar protein content from bulk oilseed sources, the NSI of dephenolized cottonseed protein (26.42–35.33%) is lower than that of peanut protein (40–55%) [48], superior to that of soybean protein (18.2–33.4%) [40] and rapeseed protein (18.73), and comparable to that of sunflower protein (20.2–37%) [67]. However, the NSI of the dephenolized cottonseed protein gradually decreases with a reduction in its free gossypol content. Generally, proteins with an NSI of ≥70% are required for optimal applications in beverages, dairy products, and other food items [68]. The current dephenolized cottonseed proteins require further modification to enhance their solubility.

#### 3.4.2. Emulsification and Emulsion Stability

Emulsification is a critical functional property of proteins that refers to their ability to form an emulsion between oil and water. Protein structure and surface hydrophobicity are intimately correlated and can directly influence the adsorption of proteins at the oil–water interface [69]. The hydrophilic and hydrophobic groups of dephenolized cottonseed protein function as surfactants to effectively reduce the interfacial tension in oil–water mixtures, thereby facilitating emulsion formation [70]. The ability to maintain a stable emulsion state without producing a two-phase separation is termed emulsification stability. The emulsification activity index (EAI) of dephenolized cottonseed protein ranges from 2.77 to 4.05 m^2^/g, which exceeds that of peanut protein (2.61 m^2^/g), is lower than that of sunflower protein (4.10 m^2^/g), and is significantly lower than that of soybean protein (14.96 m^2^/g); its emulsification stability index (ESI) is 19.17–23.14 min, which is higher than that of peanut protein (18.65 min) [71] and lower than that of soybean protein (32 min) [72] and sunflower protein (24 min) [51]. Research has demonstrated that ultrasonic and radio frequency treatments can increase the EAI and ESI of cottonseed protein to 21.17 m^2^/g and 25.06 min, and 19.61 m^2^/g and 22.12 min, respectively [73]. The emulsification properties of cottonseed protein were enhanced by 0.2 M NaCl; additionally, a specific concentration of xanthan gum and branched-chain starch can form a network structure and increase viscosity, thus improving the stability of cottonseed isolate protein emulsion [70]. Cottonseed protein isolate has a superior emulsification and emulsion stability compared to cottonseed protein concentrate, and cottonseed protein and has been successfully utilized in the production of baked products, sausages, confectionery, and other emulsified products [2].

#### 3.4.3. Foaming and Foam Stability

The foaming property refers to the capacity of a protein to form a resilient film at the gas–liquid interface, facilitating the incorporation and stabilization of a large number of bubbles. Foaming properties are typically characterized by foaming capacity (FC) and foaming stability (FS). Protein FC and FS are crucial indicators for the production of bread, cake, mousse, beer, and dairy products [65,74]. The FS of dephenolized cottonseed proteins ranges from 43.28 to 66.72%, which is comparable to that of bulk oilseed proteins; its FC ranges from 15.33 to 25.30%, which is significantly lower than that of peanut proteins (48.3%), sunflower proteins (50%), soybean proteins (63%), and rapeseed protein (89.17%) indicating that the foaming property of dephenolized cottonseed is suboptimal, necessitating further modification to achieve a competitive advantage over other bulk oilseed proteins. Currently, there is limited research on the foaming properties of cottonseed protein. Ma [2] observed that the FC (15–30%) and FS (36.8–90%) of cottonseed isolate proteins were minimal near the isoelectric point (pH 5.0), and that their foaming and stability reached a maximum at pH 7.0. However, there is a lack of research on the modification of dephenolized cottonseed protein to enhance its foaming properties.

#### 3.4.4. Water- and Oil-Holding Properties

Water-holding capacity (WHC) is a parameter that reflects the ability of proteins to absorb and retain water, whereas oil-holding capacity (OHC) represents the ability of fat particles to bind and integrate with the nonpolar side chains of proteins, which influences the juiciness, texture, and taste of food [75]. The WHC of dephenolized cottonseed protein (1.79–2.30 g/g) is lower than that of soybean proteins (3.77 g/g), rapeseed protein (3.66 g/g), and sunflower proteins (3.25 g/g), and higher than peanut protein (1.54 g/g); while its OHC (0.67–1.01 g/g) is lower than that of sunflower protein (3.78 g/g), rapeseed protein (2.63 g/g) soybean protein (1.52 g/g), and peanut protein (1.13 g/g), indicating that the water- and oil-holding properties of dephenolized cottonseed proteins are relatively poor in comparison with those of bulk oilseed proteins. The water-holding property (1.8–2.9 g/g) and oil-holding property (2.0–5.4 g/g) of dephenolized cottonseed protein isolate have been reported [76] Therefore, dephenolized cottonseed protein isolate also has the potential for application in food products such as meatballs and meat stuffing, provided that the condition of complying with the limit of free gossypol is met.

#### 3.4.5. Gelation

Protein gelation involves protein unfolding, aggregation, and aggregation association, which can form three-dimensional gel networks through hydrogen bonding, disulfide bonding, electrostatic, and hydrophobic interactions, among other mechanisms, to enhance textural properties, such as viscoelasticity and chewability in food products [77]. Favorable gelation properties can facilitate the utilization of proteins in a wide range of applications in plant-based high-protein food products, including sausages, tofu, meat, and egg analogs. The gel strength of dephenolized cottonseed protein (6.25–15.29 g) is lower than that of undephenolized cottonseed protein (25.5 g) and soy protein (15–30 g) [78]. This reduction may be attributed to the dephenolization process, in which alcoholic solvents induce the aggregation of protein molecular chains, thereby impeding the formation of gel network structures. Currently, there are few studies on the gelation of cottonseed protein at the laboratory scale or globally. Zhou Jianzhong et al. [79] found that the protein concentration, pH, and salt ions significantly influence the gel characteristics of cottonseed isolate proteins. At pH 3–7, cottonseed isolate proteins can only be formed into a gel when the concentration reaches 12%, with lower pH levels facilitating gel formation and resulting in a higher gel strength compared to high-pH conditions. Furthermore, it was observed that a high pH and low ionic strength are conducive to the formation of gels with coarse chains, large aggregates, and pores. Conversely, gels formed at a low pH and high ionic strength exhibit a fine structure with small aggregates and pores [79]. Although there is a paucity of research on the modification of dephenolized cottonseed protein to enhance its gelation properties, undephenolized cottonseed protein demonstrates favorable gelation characteristics. It offers numerous potential applications in food, such as sausages, ham, and other minced meat products, as well as cereal products, dairy products, edible packaging films, etc., provided that their utilization complies with the regulations governing the use of free gossypol as an ingredient.

In general, the functional properties of dephenolized cottonseed protein are not exceptional, and they are comparable to traditional bulk oilseed proteins in terms of solubility, water-holding capacity, and gelation properties. However, owing to the lack of fundamental research, its modification is subject to significant limitations, and there is a lack of research on the modification of dephenolized cottonseed protein to enhance its functional properties. Furthermore, the functional properties of cottonseed proteins are unstable and easily influenced by various factors (pH, etc.), resulting in the diminution of their functional attributes. This instability is one of the primary reasons for the current limitations in the development and utilization of cottonseed proteins. Compared to bulk oilseed protein, dephenolized cottonseed protein offers advantages, such as a lower cost and higher yield. Although the presence of gossypol restricts its widespread application, an increasing number of efficient technologies have been applied for cottonseed meal dephenolization. However, it is imperative to note that, in the dephenolization process, attention should be paid not only to the free gossypol content, but also to the relationship between cottonseed protein dephenolization and its functional properties. During dephenolizing, efforts should be made to retain or improve the oil holding, emulsification, foaming, and gelation functional properties of cottonseed protein to expand its application field.

**Table 6:** Comparison of functional characteristics between dephenolized cottonseed protein and bulk oilseed protein.

Type	Solubility (%)	Emulsification (m^2^/g)	Emulsion Stability (min)	Foaming Capacity (%)	Foam Stability (%)	Water- Holding Capacity (g/g)	Oil-Holding Capacity (g/g)	Gelation (g)	References
Dephenolized cottonseed protein	26.42–35.33	2.77–4.05	19.17–23.14	15.33–25.30	43.28–66.72	1.79–2.30	0.67–1.01	6.25–15.29	Laboratory self-tests
Soy protein	18.20–33.40	2.61	32.00	63.00	/	3.77	3.77	15.00–30.00	[40,72,78]
Peanut protein	40.00–50.00	14.96	18.65	48.30	/	1.54	1.13	/	[48,71]
Sunflower seed protein	20.20–37.00	4.10	24.00	50.00	/	3.25	3.78	/	[51,67]
Rapeseed protein	18.73	/	/	89.17	60.00	3.66	2.63	/	[80]

## 4. Applications of Dephenolized Cottonseed Protein

**Figure 4:**
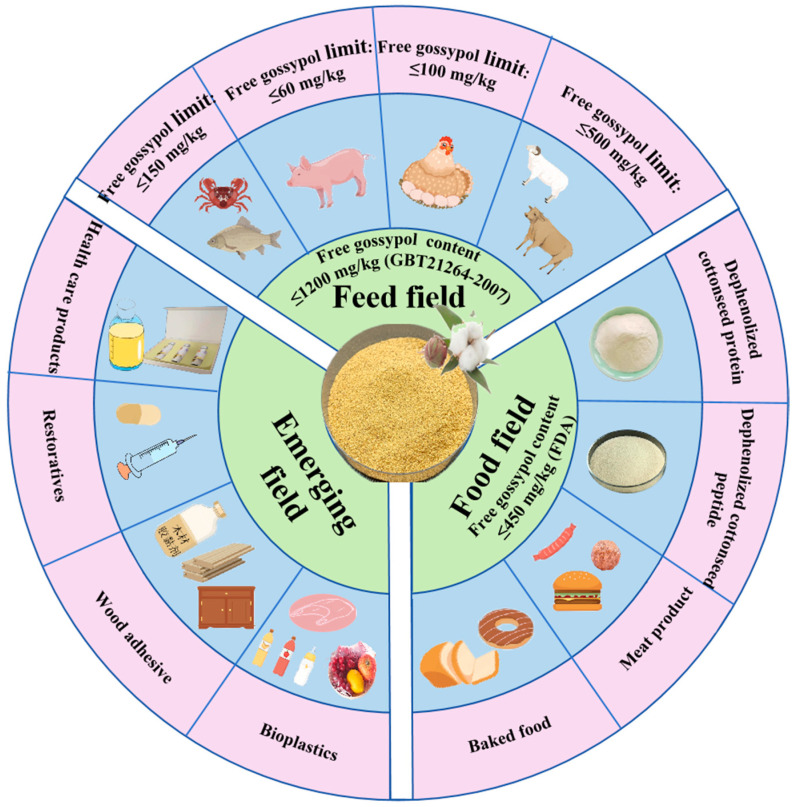
Food, feed, and emerging applications of cottonseed proteins.

With an increasing awareness of health concerns among consumers and the escalating severity of environmental issues, plant proteins have garnered significant attention in recent years as a potential substitute for animal proteins [81]. Soy protein currently dominates the plant protein market, accounting for over 50% of its value. As global population growth continues, the protein gap correspondingly expands. Traditional protein sources, such as soy and peanut, are insufficient to meet the growing demand for proteins. Consequently, researchers have focused on cottonseed protein, which offers a high yield and nutritional value. Its applications are progressively expanding into the domains of feed, food, nutraceuticals, and pharmaceuticals (Figure 4) [82].

### 4.1. Applications in Feed

Currently, China’s dephenolized cottonseed protein is primarily utilized in the feed industry (aquatic feed, swine feed, poultry feed, and ruminant feed). In 2023, the production of 50%, 55%, 60%, and 65% dephenolized cottonseed protein was 278,200, 293,800, and 44,200 tons, respectively. The 60% dephenolized cottonseed protein holds a significant position, with a market share of 49.5%. Protein fortification has emerged as a crucial strategy to enhance the nutritional value of feed products. Studies have reported that the amount of dephenolized cottonseed protein incorporated in various feeds is as follows: 20–50% in aquatic feed, 10–20% in adult pigs, 3–5% in sow feed, 10–20% in broiler feed, 5–15% in laying hen feed, 20–35% in cattle feed, and a maximum of 50% of the concentrate in sheep feed. In 2023, the market shares of 50%, 55%, 60%, and 65% dephenolized cottonseed protein for aquatic, swine, poultry, and ruminant feeds were 32.88%, 30.99%, 25.88%, and 10.25%, respectively [83,84,85]. These different feed types have varying requirements for gossypol content in dephenolized cottonseed protein, including ≤300 mg/kg for the compound feeds of herbivorous and omnivorous aquatic animals, ≤60 mg/kg for the compound feeds of pigs (except piglets) and rabbits, ≤100 mg/kg for the compound feeds of poultry (except egg-laying birds), ≤100 mg/kg for the concentrated feed supplements of calves, and ≤20 mg/kg for the compound feeds of other livestock and poultry [7]. Consequently, during application, it is imperative to adhere to the limited requirements for quality and safety indicators, such as gossypol content, as specified in existing dephenolized cottonseed protein standards (Table 7).

Fishmeal is the primary source of protein supplements for aquaculture. However, the increasing cost of fishmeal has prompted researchers to explore the use of cottonseed proteins as an alternative. Studies have found that replacing 50% of fishmeal with concentrated dephenolized cottonseed protein (CDCP) has no detrimental effects on the growth (fed for 8 weeks) and blood health of rainbow trout [64], and replacing 35.38% of fishmeal can maintain the growth performance of Chinese mitten crab [90]. The weight of black tiger shrimp can be increased by 125% with 10% CDCP protein in the feed [91]. Wang et al. [92] observed that feeding a diet supplemented with 60 g/kg dephenolized cottonseed protein in the place of fishmeal and soybean protein concentrate not only enhanced the community structure of cecal flora in weaned piglets, but also did not impair their growth performance. In broiler diets, substituting 25% soybean meal with dephenolized cottonseed protein significantly reduced daily feed intake while maintaining normal growth and development, whereas replacing 50% soybean meal with cottonseed protein concentrate diets resulted in a 7% decrease in the average daily weight gain of broilers. Studies have shown that Bahia hay treated with molasses alone or in combination with CaO fails to improve the growth performance of beef cattle. However, the addition of dephenolized cottonseed protein can be used as a protein source for beef cattle, so that they no longer lose weight [93].

The aforementioned evidence demonstrates that dephenolized cottonseed protein can serve as an alternative source of feed protein for aquatic products, livestock, and poultry, potentially reducing the dependence on soybean protein feed. It is advisable to supplement threonine, tryptophan, leucine, valine, and isoleucine in the application of dephenolized cottonseed concentrated protein, which may not only address the issue of amino acid imbalance, but also enhance its nutritional value. Concurrently, in the feed application of dephenolized cottonseed protein, attention should be directed to the contents of free gossypol, tannins, phytic acid, and other anti-nutritional factors. Moreover, the levels of aflatoxin and other pathogenic bacteria do not exceed the established standards, thereby facilitating its broader utilization in the feed industry.

### 4.2. Applications in Food

Feed-grade dephenolized cottonseed protein can be used as a raw material for the preparation of food-grade cottonseed proteins. Due to its high nutritional value and functional properties, it exhibits potential for investigation in the development of high-protein foods (such as protein powder, short peptides, food additives, protein drinks, meat substitutes, energy bars, etc.) [94]. It is anticipated to become a significant ingredient in health food innovation. To mitigate the potential health risks associated with excessive levels of gossypol, the use of dephenolized cottonseed protein should strictly adhere to existing relevant recommendations and regulatory requirements. For individuals with specific dietary needs, these products should be consumed rationally under professional guidance.

In China, the application of dephenolized cottonseed protein in food products is in its nascent stages, and the main industrial products are dephenolized cottonseed protein powder and short peptides, constituting less than 1.5% of dephenolized cottonseed protein. Research and applications in other countries have a longer history. Since the 1930s, dephenolized cottonseed protein has been used as a food additive in the United States, particularly in cookies, doughnuts, and chocolate confectioneries, with an incorporation rate of 5% [95]. In the 1970s, investigations into the use of dephenolized cottonseed protein concentrates in various food products began, including meat products (e.g., beef hamburgers, meatballs, fresh sausages, and frankfurters), extruded cereal-based products (such as snacks and plant proteins), and baked goods (e.g., cookies, doughnuts, cakes, and breads) [95]. For example, Reyes-J’aquz [96] incorporated 10% gland-free cottonseed protein powder into corn flour snacks to produce extruded snacks, which had a lower fat content than the control snacks and increased the protein content by 88%. The United States can produce concentrated protein powder with a protein content of 70% and minimal gossypol content. This product is characterized by its pure white color and absence of a distinctive odor. This cottonseed protein can be used as a meat filler and incorporated into meatballs and meat pies to reduce the gravy loss during frying and cooking and improve the balance between animal and plant proteins. Dephenolized cottonseed protein powder can also be formulated into a protein beverage that is primarily marketed in several Central American countries. In addition to cottonseed protein, this beverage contains grain flour and various vitamins, with a production cost of only one-third of that of milk. In the United States, the Fowle company authorized Bell’s Bakery to market cottonseed protein bread with a 60% higher protein content compared to standard bread. Dephenolized cottonseed protein short peptides are not only readily digestible and absorbable by the human body, but also offer antioxidant and immune-enhancing health benefits [97]. The amount added to food formulations depends on the specific requirements and nutritional needs of the target consumers. Generally, the amount of dephenolized cottonseed short peptide added as a nutrient-fortified ingredient in food can range from a few percentage points to 10% or more, with the gossypol content potentially reducing from 0.12% to 0.022% to 0.035% [96].

### 4.3. Emerging Field Applications

Despite the issue of amino acid imbalance in dephenolized cottonseed protein, feed remains its primary application. In addition to the food sector, dephenolized cottonseed proteins have recently been applied in emerging fields such as nutraceuticals, pharmaceuticals, and materials. The potential application of dephenolized cottonseed protein powder in the field of healthcare products is primarily attributed to its high protein content, low fat content, and low gossypol content. It is predominantly used for the development of functional food products, including nutritional food for pregnant women, weight loss food, and anti-fatigue food [73,98]. These products typically possess specific health benefits that can assist in regulating physiological functions and improving overall health. In nutritional supplements, dephenolized cottonseed protein powder can serve as a protein source, providing the essential amino acids and proteins necessary for a normal metabolism, bodily growth, and development. They can be used independently or in combination with other raw materials to produce compound nutritional supplements that meet the requirements of diverse populations. However, relevant reports are limited to this research stage.

The primary constituents of dephenolized cottonseed proteins in the pharmaceutical field are globulins, vitamin E, and polyphenolic compounds (e.g., flavonoids), which exhibit a wide range of bioactivities that can enhance immunity and provide nutritional benefits. Additionally, these components possess antioxidant and anti-inflammatory properties, which may contribute to the reduction in or prevention of inflammation and oxidative stress-related diseases such as cardiovascular disease and inflammatory conditions. Furthermore, they can promote wound healing, mitigate inflammatory reactions, and promote tissue repair and regeneration [94,99]. Studies have shown that the pharmaceutical field imposes stringent requirements on raw materials, necessitating the assurance of the quality and safety of dephenolized cottonseed protein to meet pharmaceutical manufacturing standards and regulatory requirements.

In addition to its applications in health care and pharmaceuticals, dephenolized cottonseed protein demonstrates potential for utilization in the production of non-feed products (including packaging, adhesives, and bioplastics). Studies have found that dephenolized cottonseed protein can be used in the manufacturing of bio- or biodegradable plastics as an alternative to traditional petrochemical plastics. This bioplastic has a favorable environmental performance and sustainability and can contribute to the reduction in environmental pollution and carbon emissions [100]. Cottonseed protein adhesive, also derived from cottonseed or dephenolized cottonseed protein, is an environmentally friendly, formaldehyde-free protein adhesive prepared using modification, cross-linking, and viscosity enhancement techniques. It possesses advantages such as a high bonding strength, nontoxicity, tastelessness, and low cost [101]. Under equivalent preparation process conditions, the adhesive strength of cottonseed protein adhesive is significantly better than that of soybean protein and peanut meal protein adhesives, while maintaining the lowest cost among the three. This suggests a considerable potential for application development, although industrial-scale production has not yet been realized [102]. De Oliveira Filho [103] developed an alginate film incorporating cottonseed protein hydrolysate, which shows promise as an active packaging material for the preservation of fatty foods.

The aforementioned evidence suggests that dephenolized cottonseed protein has potential for application in the domains of healthcare products, pharmaceuticals, and materials. However, since dephenolized cottonseed protein still contains a certain amount of free gossypol, its application should be considered, particularly in the fields of food, healthcare products, and pharmaceuticals. As the scientific interest in dephenolized cottonseed protein continues to increase, it is anticipated that its applications will become more extensive and thoroughly explored in future research.

## 5. Conclusions and Future Prospects

This paper presents a detailed review and introduction of recent advancements in gossypol removal methods and the quality characteristics and application domains of dephenolized cottonseed protein. Moreover, this review discusses dephenolization methods, including the optimization and improvement of physical, chemical, and biological approaches, as well as novel techniques such as employing gene silencing technology to reduce the free gossypol in cottonseed and cultivating glandless cotton varieties, among other strategies. In terms of quality characteristics, this review elucidates not only the fundamental composition, structural characteristics, and functional properties of dephenolized cottonseed protein, but also explores its similarities and differences with bulk oilseed protein through comparative analysis. In addition, the application of dephenolized cottonseed protein in the emerging fields of feed, food, and health products, medicine, and materials is systematically discussed.

In recent years, the plant protein industry has experienced rapid development. As a new plant protein resource, dephenolized cottonseed protein has shown great value in solving the global protein resource shortage and realizing the high-value utilization of agricultural by-products. The application of dephenolized cottonseed protein in different fields faces numerous challenges, owing to limitations in gossypol content and the functional properties of the protein. However, with technological advancements and further expansion of the plant protein market, the dephenolized cottonseed protein industry has encountered new development opportunities. Regarding future research prospects, the following aspects warrant attention: (1) the existing free gossypol removal process needs to be further optimized, and it is urgent to explore a simple, green, efficient, and economical method for the removal of gossypol and build an online detection system for gossypol; (2) the analysis of the quality characteristics of dephenolized cottonseed protein is not systematic enough, particularly in the quality analysis of different varieties of dephenolized cottonseed protein; (3) the modification of dephenolized cottonseed protein to enhance its functional properties requires urgent investigation, and its modification mechanism to be elucidated; (4) the effect of dephenolized cottonseed protein in feed and food applications necessitates systematic study, especially the relationship between its dosage and product quality, and its safety requires further evaluation; and (5) there are a lack of relevant standards for the application of dephenolized cottonseed protein in food, health products, and medicine. With increasing attention on dephenolized cottonseed protein and active exploration and breakthroughs by researchers in science and technology, dephenolized cottonseed protein is expected to become an important component of the protein supply system and will have wide application prospects in the near future.

## Figures and Tables

**Table 1:** Comparative analysis of basic physical and chemical indicators of dephenolized cottonseed protein and bulk oilseed protein.

Type	Moisture (%)	Ash (%)	Crude Fiber (%)	Crude Protein (Feed) (%)	Crude Protein (Food) (%)	Crude Fat (%)	Total Sugar (%)	References
Cottonseed protein	3.26–7.78	7.30–7.50	4.20–5.85	57.10–61.50	48.20–52.40	1.50–1.85	1.10–1.55	Laboratory self-tests
Soy protein	8.23	5.44	4.13	/	56.41	3.17	1.14	[40]
Peanut protein	6.72	5.11	8.78	/	53.87	7.74	2.33	[41,42]
Sunflower protein	4.29	5.90	7.50	/	47.10	9.80	1.02	[43]

Note: 1. All proteins in the table refer to protein powder; 2. Crude protein (feed) is measured according to the national standard for feed [44], with a protein coefficient of 6.25; crude protein (food) is measured according to the national standard for food (GB 5009.5-2016) [45], with a protein coefficient of 5.3; 3. Total sugar is calculated as the sum of fructose, glucose, sucrose, maltose and lactose.

**Table 3:** Comparative analysis of amino acid content of dephenolized cottonseed protein and bulk oilseed protein.

Amino Acids (g/16 g N)	Cottonseed Protein	Soy Protein	Peanut Protein	Sunflower Seed Protein
Lysine (Lys) *	4.4	6.9	3.0	3.0
Histidine (His) *	2.9	2.6	2.4	2.5
Arginine (Arg)	12.4	8.4	12.6	7.9
Aspartic acid (Asp)	9.1	12.0	12.5	9.1
Threonine (Thr) *	3.0	4.3	2.5	3.7
Serine (Ser)	4.1	5.6	5.2	3.6
Glutamic acid (Glu)	20.4	21.0	20.7	20.5
Proline (Pro)	3.6	6.2	4.6	4.0
Glycine (Gly)	4.1	4.6	4.2	4.0
Alanine (Ala)	3.7	4.5	4.0	4.1
Cystine	0.8	1.6	1.4	1.1
Valine (Val) *	4.6	5.3	4.5	4.6
Methionine (Met) *	1.3	1.5	1.0	2.0
Isoleucine (Ile) *	3.4	5.1	3.4	3.9
Leucine (Leu) *	5.8	7.7	6.7	6.9
Tyrosine (Tyr)	3.1	3.9	4.4	2.5
Phenylalanine (Phe) *	5.5	5.0	5.6	5.6
Tryptophan (Trp) *	1.1	1.2	1.0	1.0
Essential amino acid	26.5	39.6	30.1	33.2

Note: Those marked with * in the table are essential amino acids.

**Table 4:** Vitamin content of dephenolized cottonseed protein in different enterprises.

Vitamins (μg/100 g)	New Race Protein	Dawn Protein	Tiankang Protein	Jinlan Protein	Taikun Protein
α-Tocopherol	300	347	342	425	274
β-Tocopherol	<120	<120	<120	<120	<120
γ-Tocopherol	1840	1920	1460	1790	1710
δ-Tocopherol	<120	<120	<120	<120	<120
Vitamin B1	394	180	342	425	467
Vitamin A	<30	<30	<30	<30	<30
Vitamin D	<0.6	<0.6	<0.6	<0.6	<0.6
Vitamin E	2130	2280	1810	2080	1970

**Table 7:** Requirements for standard quality and safety indicators for feed dephenolized cottonseed protein (the following dephenolized cottonseed protein standards are Chinese standards).

Standard	Project		Excellent	Level 1	Level 2	Level 3
GH/T 1042-2007 [86]	Crude protein %		≥50.0	≥50.0	≥48.0	—
	Crude fiber %		≤7.5	≤8.0	≤9.0	
	Moisture %		≤8.0	≤8.0	≤12.0	
	Crude ash %		≤8.0	≤8.5	≤9.0	
	Total amino acids %		≥90.0	≥87.0	≥85.0	
	Free gossypol %	Liquid chromatography	≤0.006	≤0.006	≤0.010	
		Spectrophotometric method	≤0.040	≤0.040	≤0.055	
	Aflatoxin B1 (µg/kg)		≤50	≤50	≤50	
	Total mold count (cfu/g)		<50 × 10^3^	<50 × 10^3^	<50 × 10^3^	
	*Salmonella*		not detectable	not detectable	not detectable	
DB43/T 885-2014 [87]	Crude protein %		——	≥50.0	≥50.0	—
	Crude fiber %		——	≤7.5	≤9.5	
	Moisture %		——	≤8.0	≤9.0	
	Crude ash %		——	≤7.0	≤8.0	
	Percentage of amino acids in crude protein %		——	≥88.0	≥88.0	
	Free gossypol %		——	≤0.040	≤0.040	
	Crude protein %		——	≥50.0	≥50.0	
	Aflatoxin B1 (µg/kg)		≤50	≤50	≤50	
	Total mold count (cfu/g)		<50 × 10^3^	<50 × 10^3^	<50 × 10^3^	
	*Salmonella*		not detectable	not detectable	not detectable	
Q/XJDT001-2019 [88]	Crude protein %		≥60.0	≥55.0	≥50.0	—
	Crude fiber %		≤5.0	≤7.0	≤8.0	
	Moisture %		≤8.0	≤8.0	≤12.0	
	Crude ash %		≤7.0	≤7.0	≤9.0	
	Percentage of amino acids in crude protein %		≥90	≥90	≥87	
	Free gossypol (mg/kg)		≤400	≤400	≤400	
	Aflatoxin B1 (µg/kg)		≤40	≤40	≤40	
	Total mold count (cfu/g)		<50 × 10^3^	<50 × 10^3^	<50 × 10^3^	
	*Salmonella*		not detectable	not detectable	not detectable	
Q/XJJL001-2017 [89]	Crude protein %		≥60.0	≥55.0	≥53.0	≥50.0
	Crude fiber %		≤7.0	≤7.0	≤7.0	≤8.0
	Moisture %		≤8.0	≤8.0	≤8.0	≤12.0
	Crude ash %		≤7.0	≤8.0	≤9.0	≤9.0
	Percentage of amino acids in crude protein %		≥87	≥87	≥87	≥87
	Free gossypol (mg/kg)		≤400	≤400	≤400	≤400
	Aflatoxin B1 (µg/kg)		≤40	≤40	≤40	≤40
	Total mold count (cfu/g)		<50 × 10^3^	<50 × 10^3^	<50 × 10^3^	<50 × 10^3^
	*Salmonella*		not detectable	not detectable	not detectable	not detectable

## Data Availability

No new data were created or analyzed in this study.
